# Subacute Combined Degeneration as a Red Herring for Acute Traumatic Spinal Cord Injury

**DOI:** 10.7759/cureus.115041

**Published:** 2026-08-23

**Authors:** Dylan L McCaleb, Rebecca Stewart, Aileen Giordano

**Affiliations:** 1 Physical Medicine and Rehabilitation, University of Virginia Health System, Charlottesville, USA; 2 Physical Medicine and Rehabilitation, University of Virginia School of Medicine, Charlottesville, USA

**Keywords:** b12 deficiency, nitrous oxide, orthostatic hypotension, subacute combined degeneration, traumatic spinal cord injury

## Abstract

Subacute combined degeneration (SCD) is a potentially reversible neurologic disorder caused by vitamin B12 deficiency that classically affects the dorsal columns of the spinal cord. Clinical manifestations include sensory loss, gait instability, weakness, and, less commonly, autonomic dysfunction. With these neurologic manifestations, patients may present with recurrent falls, and SCD can mimic the presentation of acute traumatic spinal cord injury (SCI).

In this case, a 66-year-old man with a recent history of nitrous oxide use presented with bilateral lower extremity weakness, sensory changes, and gait instability, resulting in recurrent ground-level falls. Initial evaluation for traumatic SCI was unrevealing, and the patient was discharged home. Due to continued symptom progression, the patient presented again and underwent further workup that demonstrated significant B12 deficiency secondary to pernicious anemia. Cervical MRI revealed symmetric dorsal column T2 hyperintensity in the axial plane forming the “inverted V" sign that is characteristic of SCD. Despite serum B12 level correction, the patient had persistent neurologic deficits requiring admission to inpatient rehabilitation.

This case highlights SCD as an important diagnostic consideration in the setting of acute trauma and emphasizes the importance of patient risk factors such as nitrous oxide abuse. Accurate diagnosis is contingent on recognition of key imaging findings and pursuit of further laboratory workup, especially when initial traumatic workup is negative. Early diagnosis is critical as delayed treatment may result in irreversible neurologic impairment and functional disability.

## Introduction

Subacute combined degeneration (SCD) is a neurologic condition secondary to vitamin B12 deficiency that leads to demyelination of the dorsal columns of the spinal cord. Patients most commonly present with loss of proprioception and vibration sensation, paresthesia, ataxia, gait impairment, and weakness. In more severe cases, involvement of the autonomic nervous system may lead to dysautonomia, resulting in significant orthostatic hypotension that can worsen functional impairments [1,2].

Vitamin B12 deficiency results from a variety of etiologies, including pernicious anemia, nutritional deficiency, chronic alcohol or inhalant abuse, and medication side effects. Nitrous oxide specifically has been shown to cause vitamin B12 deficiency as it irreversibly oxidizes cobalamin, inhibits its biologic activity, and leads to demyelination of neurons [2,3].

The clinical presentation of SCD can often overlap with acute traumatic spinal cord injury, especially in patients who present after falls. Distinguishing between traumatic and metabolic causes of neurologic deficits can be challenging but is essential due to differences in management strategies and prognostication. MRI plays a significant role in diagnosing SCD with characteristic axial plane symmetric dorsal column T2 hyperintensity as the hallmark finding of this process [1,4].

We report a case of nitrous oxide abuse and subsequent SCD in a patient found to have pernicious anemia after initial workup for acute traumatic SCI was unremarkable. This case highlights the importance of patient history and creating a broad differential diagnosis when treating patients who present with falls and acute neurologic deficits. Further laboratory workup and MRI findings were essential in identifying a treatable, non-traumatic etiology of the patient’s presentation.

## Case presentation

We present a case of a 66-year-old man with a history of alcohol and inhalant abuse who developed bilateral lower extremity weakness, paresthesia, and gait ataxia following multiple ground-level falls in the setting of significant nitrous oxide use two weeks earlier. He initially presented to the emergency department with concern for acute traumatic spinal cord injury (SCI). Non-contrast CT of the head and cervical spine was unremarkable for an acute process at the initial emergency department visit, and the patient was discharged home without further workup. Worsening weakness, progressive sensory loss to the mid-thoracic level, and sensory gait ataxia led to his re-presentation 10 days later to the emergency department, prompting further workup.

On re-presentation to the emergency department, physical examination of the patient demonstrated loss of vibration sensation to the mid-thoracic region. The patient had bilateral lower extremity weakness with 3-4/5 strength grading throughout. He demonstrated ataxia with difficulty on finger-to-nose and heel-to-shin testing as well as increased tone and hyporeflexia in all extremities. An American Spinal Injury Association (ASIA) exam was not performed in the acute care setting with limited prognostic value in a non-traumatic SCI case. Orthostatic vital signs showed a 46 mmHg drop in systolic blood pressure from supine (110/57 mmHg) to standing (64/48 mmHg).

**Figure 1:**
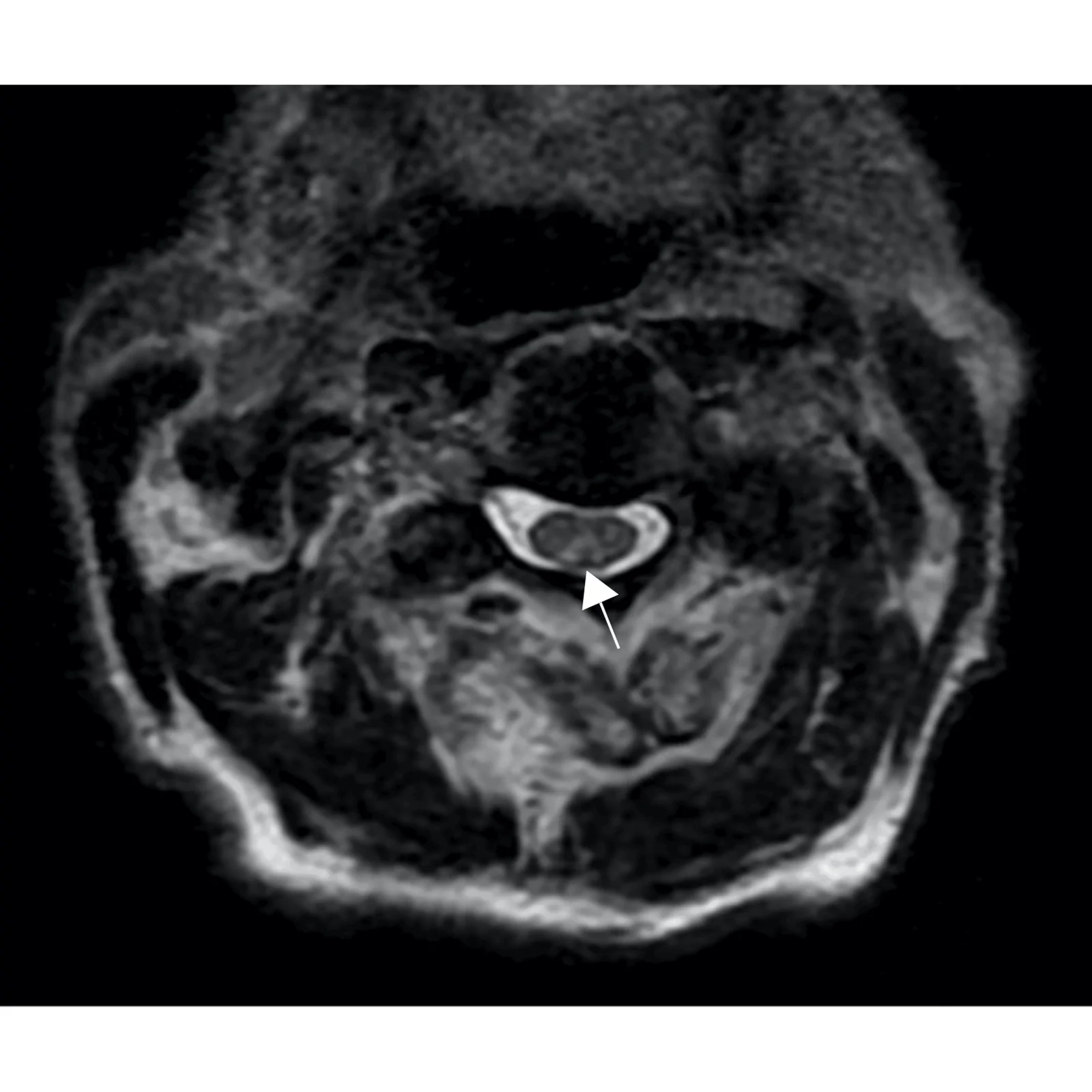
Magnetic resonance imaging (MRI) of the cervical spine Axial view-T2 sequence revealed a bilaterally symmetric hyperintensity in the cervical dorsal columns, forming the characteristic "inverted V" consistent with subacute combined degeneration.

Laboratory evaluation revealed a vitamin B12 level <150 pg/mL (normal reference: 200 to 900 pg/mL) and macrocytic anemia with mean corpuscular volume (MCV) 103 fL (normal reference: 80 to 100 fL). Intrinsic factor antibodies were positive, consistent with a diagnosis of pernicious anemia. Additionally, MRI of the cervical spine demonstrated symmetric T2 hyperintensity of the dorsal columns in the axial plane, forming the characteristic "inverted V" sign that is the hallmark of SCD (Figure 1) [1,4]. Intramuscular vitamin B12 injections were initiated; however, the patient’s sensory loss, ataxia, and orthostasis persisted despite normalization of serum B12 levels, as is commonly described in SCD [1]. Due to persistent neurologic deficits and orthostasis, the patient was discharged to an inpatient rehabilitation facility (IRF) for functional optimization. The patient presented to IRF at a dependent level with any ambulation. He completed a 12-day course of intensive rehabilitation and was able to ambulate 200 feet with the use of a 4-pronged walker with only contact-guard assist on discharge.

## Discussion

In this case, it is important to maintain a broad differential for these neurologic symptoms. Diagnostic considerations for this presentation should include but are not limited to acute traumatic spinal cord injury, cervical myelopathy, transverse myelitis, spinal cord ischemia, multiple sclerosis, and metabolic derangements. Vitamin B12 deficiency is a relatively uncommon cause of these symptoms compared to other considerations, with a prevalence of 2-3% in the United States [5]. Furthermore, among patients diagnosed with B12 deficiency, it has been shown that 25% had symptoms of peripheral neuropathy and only 11% had SCD [6].

Inadequate vitamin B12 leads to the accumulation of myelotoxic methylmalonic acid, which preferentially affects long, heavily myelinated tracts like the dorsal columns, with relative sparing of the tracts in the anterior spinal cord, leading to the characteristic imaging finding in SCD--the "inverted V sign" [4]. Of note, nitrous oxide can be a precipitating factor for functional B12 deficiency. Nitrous oxide irreversibly oxidizes and inactivates cobalamin, which is particularly harmful in patients with impaired B12 stores due to underlying pernicious anemia and chronic alcohol abuse, and can trigger SCD [2,3].

Although characteristically associated with sensory deficits, motor deficits can be seen in SCD with more prolonged or severe disease [7]. The coexistence of increased tone (an upper motor neuron sign) and hyporeflexia (a lower motor neuron sign) noted in this case results from concurrent central and peripheral demyelination seen in B12 deficiency myeloneuropathy [1].

Similarly, although significantly less common than sensory manifestations, autonomic dysfunction is a clinically significant manifestation of SCD with important implications for rehabilitation [2,8]. Orthostatic hypotension is a rarely reported manifestation of SCD, largely discussed in isolated case reports [9]. In this patient, orthostatic hypotension was initially a significant barrier to participation in therapy, and effective medical management of this uncommon symptom of SCD was essential for safe mobilization and functional progress in the IRF. Orthostasis was successfully treated in IRF with midodrine 15 mg 3 times daily along with the use of compression stockings and an abdominal binder when out of bed. He progressed very well in the IRF and was able to transition home on this regimen at a modified independent level for household and community mobility with use of a walker.

This case demonstrates the diagnostic challenge that patients who present with recurrent falls, bilateral neurologic deficits, and upper motor neuron signs present. Specifically, while this presentation is consistent with a cervical SCI, it is often difficult to work out if recurrent falls are the cause of the SCI (as in a traumatic cervical SCI) or a symptom of the SCI (as in SCD). Recognition of characteristic MRI findings is essential for prompt identification of underlying causes of myelopathy following trauma, which can be critical to guiding treatment. In this case, a key imaging finding, the "inverted V" sign, in conjunction with a thorough history, exam, and laboratory workup, confirmed an unexpected, treatable, non-traumatic etiology for the patient’s symptoms, specifically, SCD.

Additionally, this case demonstrates that SCD symptoms can extend beyond common sensory deficits and can include motor and autonomic deficits. Thus, SCD should remain in the differential when a patient presents with unexplained ataxia, even if concurrent motor or autonomic symptoms are present [7,9].

## Conclusions

Subacute combined degeneration (SCD) should be a consideration in the differential diagnosis of patients presenting with recurrent falls and neurologic deficits, particularly when risk factors for vitamin B12 deficiency are present. This case illustrates how SCD can mimic acute traumatic spinal cord injury, which can lead to a diagnostic delay. In addition to thorough history taking and laboratory workup, recognition of characteristic MRI findings, such as the “inverted V’ sign, is essential for identifying the diagnosis. Early recognition of precipitating factors for vitamin B12 deficiency, such as nitrous oxide use, leads to the timely diagnosis and treatment of SCD, which, in turn, may reduce the progression of neurologic deficits and provide the opportunity to implement early rehabilitative interventions to maximize the patient’s functional recovery.

## References

[1] Kumar N (2014). Neurologic aspects of cobalamin (B12) deficiency. Handb Clin Neurol.

[2] Healton EB, Savage DG, Brust JC, Garrett TJ, Lindenbaum J (1991). Neurologic aspects of cobalamin deficiency. Medicine (Baltimore).

[3] Agbo SM, Duan X, Wang L, Dong M, Wang Q, Cai X, Liu F (2025). Comparative study of subacute combined degeneration of the spinal cord due to nitrous oxide abuse and vitamin B12 deficiency. Front Immunol.

[4] Kumar A, Singh AK (2009). Teaching NeuroImage: inverted V sign in subacute combined degeneration of spinal cord. Neurology.

[5] Patel H, McGuirk R (2025). Vitamin B12 deficiency: common questions and answers. Am Fam Physician.

[6] Reynolds E (2006). Vitamin B12, folic acid, and the nervous system. Lancet Neurol.

[7] Bao L, Li Q, Li Q, Chen H, Zhang R, Shi H, Cui G (2020). Clinical, electrophysiological and radiological features of nitrous oxide-induced neurological disorders. Neuropsychiatr Dis Treat.

[8] Robert Harker DM, Martinez B, Tabaac BJ (2021). B12 deficiency and clinical presentation in the setting of nitric oxide use. Case Rep Neurol Med.

[9] Toru S, Yokota T, Inaba A, Yamawaki M, Yamada M, Mizusawa H, Hayashi M (1999). Autonomic dysfunction and orthostatic hypotension caused by vitamin B12 deficiency. J Neurol Neurosurg Psychiatry.

